# Development of a Wine Yeast Strain Capable of Malolactic Fermentation and Reducing the Ethyl Carbamate Content in Wine

**DOI:** 10.3390/foods14010054

**Published:** 2024-12-27

**Authors:** Egor A. Vasyagin, Valery N. Urakov, Maksim Yu. Shalamitskiy, Sofia N. Cherviak, Elena V. Ivanova, Valentina I. Zagoruyko, Alexey V. Beletsky, Andrey L. Rakitin, Eugenia S. Mardanova, Vitaly V. Kushnirov, Nikolai V. Ravin, Andrey V. Mardanov

**Affiliations:** 1 Institute of Bioengineering, Research Center of Biotechnology of the Russian Academy of Sciences, 119071 Moscow, Russiarakitin@biengi.ac.ru (A.L.R.);; 2 Bach Institute of Biochemistry, Research Center of Biotechnology of the Russian Academy of Sciences, 119071 Moscow, Russia; valery.urakov@gmail.com (V.N.U.);; 3 All-Russian National Research Institute of Viticulture and Winemaking “Magarach”, Russian Academy of Sciences, 298600 Yalta, Russia; mshalamitskiy@yahoo.com (M.Y.S.); sofi4@list.ru (S.N.C.); lenochka_ivanova_58@mail.ru (E.V.I.); valya.yalta64@mail.ru (V.I.Z.)

**Keywords:** wine yeast, malolactic fermentation, ethyl carbamate, *Saccharomyces cerevisiae*, CRISPR/Cas9, genome editing

## Abstract

In winemaking, malolactic fermentation (MLF), which converts L-malic acid to L-lactic acid, is often applied after the alcoholic fermentation stage to improve the sensory properties of the wine and its microbiological stability. MLF is usually performed by lactic acid bacteria, which, however, are sensitive to the conditions of alcoholic fermentation. Therefore, the development of wine yeast strains capable of both alcoholic fermentation and MLF is an important task. Using genome editing, we engineered a modified variant of the triploid wine yeast strain *Saccharomyces cerevisiae* I-328, in which the *CAR1* arginase gene was replaced by the malate permease gene from *Schizosaccharomyces pombe* and the malolactic enzyme gene from *Oenococcus oeni*. Genome-wide transcriptional profiling confirmed the expression of the introduced genes and revealed a limited effect of the modification on global gene expression. Winemaking experiments show that genome editing did not affect fermentation activity and ethanol production, while use of the modified strain resulted in a tenfold reduction in malate content with simultaneous formation of lactate. The resulting wines had a softer and more harmonious taste compared to wine obtained using the parental strain. Inactivation of arginase, which forms urea and L-ornithine through the breakdown of arginine, also resulted in a twofold decrease in the content of urea and the carcinogenic ethyl carbamate in wine. Thus, the new strain with the replacement of the arginase gene with the MLF gene cassette is promising for use in winemaking.

## 1. Introduction

The vast majority of red wines, as well as some white and sparkling wines, undergo two consecutive stages of fermentation: alcoholic fermentation (AF) and malolactic fermentation (MLF), in which L-malic acid (malate) is converted to L-lactic acid (lactate) with the release of carbon dioxide [1]. The content of malic acid in wine in an amount of more than 2 g/L causes a disturbance of taste and imparts an unpleasant tint of “green acidity”. It may also promote spontaneous MLF, which creates the risk of formation of elevated concentrations of volatile acids and biogenic amines, which negatively affects the quality of the wine [2,3]. Incorporating MLF into the production cycle reduces wine acidity and enhances its sensory attributes, such as buttery, fruity, or nutty aromas, while also improving microbiological stability [4,5]. Typically, MLF is carried out by lactic acid bacteria (LAB) in the wine, such as *Oenococcus oeni*. However, these bacteria are sensitive to the inhibitory conditions of alcoholic fermentation, which include low pH, high ethanol concentration, and the presence of SO_2_ [6,7]. Therefore, a wine yeast strain capable of performing both AF and MLF simultaneously would be of great interest to winemakers. Previously, a strain of *Saccharomyces cerevisiae* ML01 [8] was described that is capable of simultaneously conducting AF and MLF. This strain, derived from the wine yeast strain S92 using genetic engineering, contains the *MAE1* malate permease gene from the yeast *Schizosaccharomyces pombe* and the *mleA* malolactic enzyme gene from the bacterium *O. oeni*, under the control of the strong constitutive promoter of the *S. cerevisiae PGK1* gene. The malolactic cassette was integrated into the *URA3* locus. The strain can completely ferment malic acid at a concentration of 5.5 g/L in must within 5 days, without negatively affecting the wine’s sensory qualities. Phenotypic, transcriptomic, and proteomic analyses showed that the ML01 strain has minor differences from the parental S92 strain. ML01 was the first strain of wine yeast with a genetically modified genome that received Generally Recognized as Safe (GRAS) status from the U.S. Food and Drug Administration and was used in the wine industry [8,9].

An important problem in the production of fermented products, particularly wine and stronger beverages, is the accumulation of ethyl carbamate (EC) in them. EC is a Group 2A carcinogen [10]. EC can accumulate in wine and winemaking products over time, and many countries strictly regulate the maximum allowable content of this substance in food products [11]. However, there is currently no general standard that regulates the maximum allowable level of ethyl carbamate in wine. For example, the maximum allowable concentration of ethyl carbamate in wine in Canada and the Czech Republic is 30 μg/L, while in the USA it is legally regulated at 15 μg/L.

EC is formed in wine as a result of the interaction between ethanol and urea excreted by yeast cells. Urea, in turn, is produced together with L-ornithine through the breakdown of arginine by the Car1 arginase, encoded by the *CAR1* gene. Arginine is sourced from must, where it is present at high concentrations. The problem is that removing ethyl carbamate from wine distillates is a complex and costly process. Therefore, a more promising approach is to reduce the accumulation of this compound by applying yeast metabolic engineering methods (reviewed in [12]). One such method involves decreasing arginase activity through the deletion or inhibition of the *CAR1* gene, which is involved in the production of urea, a precursor of EC [13,14,15]. Another method focuses on the overexpression of the *DUR1,2*, and *DUR3* genes, which are involved in urea degradation and transport [16]. Mutations in the *CAN1* and *GAP1* genes, which regulate arginine transport into yeast cells, have also been shown to reduce urea and EC levels [17,18]. Additionally, modulating the expression of four GATA transcription factors (Gln3p, Gat1p, Dal80p, and Gzf3p) that regulate nitrogen catabolite repression-sensitive genes effectively lowers extracellular urea and EC concentration [19]. Finally, the simultaneous deletion of the *CAR1* and *GZF3* genes reduced EC content by up to 52% [20].

In this work, we aimed to create an engineered strain of wine yeast that could simultaneously carry out MLF and produce smaller amounts of urea and EC. The wine yeast strain *S. cerevisiae* I-328 from The Magarach Collection of Microorganisms for Winemaking was used for genetic modification via the CRISPR/Cas9 system. To enable this strain to carry out MLF while reducing EC production, we integrated the MLF expression cassette in place of the *CAR1* gene. The genes *mleA* and *MAE1* were sourced from *O. oeni* and *S. pombe*, respectively. The impact of *CAR1* gene deletion and MLF cassette insertion in the *S. cerevisiae* I-328 strain was investigated using genomic and transcriptomic approaches. The performance of the engineered strain in winemaking was evaluated.

## 2. Materials and Methods

### 2.1. Strains, Media, and Cultivation Conditions

The *Escherichia coli* DH10B strain was grown at 37 °C in Luria–Bertani (LB) medium. The DH10B strain was employed to maintain plasmids used for genetic engineering. The *S. cerevisiae* strain I-328 deposited in the Magarach Collection of Microorganisms for Winemaking [21,22] was used as a host for the integration of the MLF cassette and *CAR1* deletion. Yeasts were cultured aerobically with shaking at 30 °C either in YPD medium (1% yeast extract, 2% peptone, 2% glucose) or SC media (0.17% yeast nitrogen base without ammonium sulfate, 2% glucose, and indicated amounts of acetamide (SC + acet) or arginine (SC + arg) as a nitrogen source). Transformation of yeast was performed using the lithium acetate method, following the protocol described by Gietz and Woods [23].

### 2.2. Construction of a MLF Gene Cassette for Integration into the Genome of Yeast Strain I-328

All oligonucleotides and plasmids used in this study are summarized in Table 1 and Table 2. All PCR amplifications were performed using Phusion polymerase (Thermo Fisher Scientific, Waltham, MA, USA). PCR fragments were separated by agarose gel electrophoresis and purified using the QIAquick Gel Extraction Kit (Qiagen, Heiden, Germany).

At the first stage, a fragment containing flanking sequences of the *CAR1* gene was created for homologous recombination in strain *S. cerevisiae* I-328 [22]. The first 220 bp long fragment (Car1_2), located in the intergenic space upstream of the *CAR1* gene, was amplified using primers Car_1F/Car_2R (Table 1). The second fragment (Car3_4) was 348 bp in size and located in the intergenic space downstream of the *CAR1* gene. It was amplified using primers Car_3F/Car_4R (Table 1). The obtained fragments were combined into a single 586 bp delCar1_4 fragment using overlap extension PCR (OE PCR). Restriction sites *Xma*I and *Xho*I were inserted in the middle of the delCar1_4 fragment. The resulting fragment was cloned to the *Sma*I site of the pUC19 vector; thus, the plasmid pUC_delCar1_4 was obtained (Table 1).

In the next step, the *mleA* gene encoding malolactic enzyme from the lactic acid bacterium *O. oeni* K19-3 [24] was amplified using the mleF/mleR primer pair. Additionally, the Ppgk1 promoter and Tpgk1 terminator of the *PGK1* gene were amplified from the genomic DNA of *S. cerevisiae* I-329 [25] using the primer pairs PpgkF/ PpgkR and TpgkF/TpgkR, respectively. The obtained three PCR fragments were assembled by OE PCR with primers PpgkF and TpgkR and then cloned into the *Nru*I/*Xho*I sites of pUC19_delCar1_4 plasmid, resulting in plasmid pUC19_delCar1_4_Mle.

At the final stage, the *MAE1* gene with its own terminator sequence was amplified from the genomic DNA of *S. pombe* strain I-583 using the primers maeF/maeR. The Ptdh promoter was amplified from the genomic DNA of *S. cerevisiae* I-329 using the primers PtdhF/ PtdhR. The resulting fragments were assembled using OE PCR with primers maeF and maeR to form the Tmae-mae1-Ptdh cassette, which was cloned into the pUC19_delCar1_4_Mle plasmid at the *Nru*I/*Xma*I sites, resulting in the pUC19_delCar1_4_MLF plasmid.

To obtain a linear donor DNA fragment for genome editing, the plasmid pUC19_delCar1_4_MLF was cleaved by restriction enzymes *Msc*I and *Eco*47III. The resulting 5078 bp long DNA fragment contained the MLF gene cassette flanked by regions required for integration into the *S. cerevisiae* I-328 genome instead of the *CARI* gene by homologous recombination.

### 2.3. Genome Editing

Genome editing was performed using a modified technique based on the CRISPR/Cas9 yeast system described earlier [26]. Two vectors were used to make a double-stranded cut in the *CAR1* genomic locus, one of which, pWS-amdS, encodes the Cas9 nuclease and also contains a selective amdS marker that ensures the growth of yeast transformants in SC medium with acetamide as a nitrogen source [27]. The second vector, pWS82-CAR1-3, encodes the sgRNA necessary to target the Cas9 nuclease to the *CAR1* gene [27]. The strain I-328 was simultaneously transformed by the *Esp*3I fragment (10.3 kb) of the pWS-amdS vector and the *Eco*RV fragment (2046 bp) of the pWS82-CAR1-3 targeting plasmid, as well as the donor fragment (5078 bp) containing the MLF gene expression cassette and flanked by regions required for replacing the *CAR1* locus via homologous recombination. Transformation was carried out as previously described [23]. Transformants were selected on a solid SC+acet solid medium containing acetamide (0.3%) instead of ammonium sulphate [27].

### 2.4. Genome Sequencing and Assembly

A single colony of *S. cerevisiae* I-328 ∆CAR1_MLF was grown in 5 mL of YPD medium at 20 °C for 24 h; then, cells were collected by centrifugation at 4000× g for 5 min. Total DNA was isolated using the DNeasy PowerSoil Kit (Qiagen, Hilden, Germany) following the manufacturer’s protocol. The genome of *S. cerevisiae* I-328 ∆CAR1_MLF strain was sequenced using Illumina technology (Illumina, San Diego, CA, USA). Paired-end DNA libraries were prepared using the NEBNext^®®^ Ultra™ II DNA Library Prep Kit (NEB, Ipswich, MA, USA) in accordance with the manufacturer’s instructions. The sequencing of this library generated 1,099,714 paired-end reads (2 × 300 nt). Sequencing primers were removed using Cutadapt v.4.0 [28] and low quality read regions were trimmed using Sickle v.1.33 [29]. Illumina reads were assembled into contigs using SPAdes v.3.15.4 in isolate mode.

Illumina reads were also mapped on reference genome of the parental strain *S. cerevisiae* I-328 (GeneBank PEJR00000000) using Bowtie 2 v.2.3.5.1 [30]. Freebayes v.1.2.0-4 [31] was used to find SNPs. Fractions of reads supporting SNPs were visualized as a histogram in R to identify ploidy of the genome. Mapping of I-328 reads with Bowtie 2 on I-328 ∆CAR1_MLF contigs confirmed the insertion of *mleA* and *MAE1* genes.

### 2.5. Transcriptome Sequencing and Analysis of Gene Expression

To analyze the transcription profile of the *S. cerevisiae* I-328 ∆CAR1_MLF strain under microvinification conditions, total RNA was isolated from the yeast culture. The parent strain *S. cerevisiae* I-328 was used as a control. The cultures of I-328 ∆CAR1_MLF and I-328 strains were grown in 5 mL of must from the Aligoté grape variety at 28 °C for 72 h. The cultures were then transferred into 100 mL of must and grown at 28 °C for 72 h. To simulate winemaking conditions, the cultures were transferred to 1 L of must and grown at 20 °C for 96 h. Then, 10 mL was taken from each culture to isolate total RNA. RNA extraction was performed using the hot phenol method [32], followed by purification with the RNeasy Mini Kit (Qiagen, Hilden, Germany). The preparation of mRNA libraries was performed using the TruSeq RNA Library Prep Kit v2, following the manufacturer’s protocol (Illumina, San Diego, CA, USA). Sequencing was carried out on the Illumina HiSeq platform (Illumina, San Diego, CA, USA), generating 18 to 40 million 50 nt single-end reads per sample. The expression analysis used the previously assembled genome of strain I-328 [GenBank PEJR00000000, [22]] as a reference with the addition of *mleA* (WP_002823502) and *MAE1* (POMI540_4738) gene sequences.

Gene expression levels were estimated using RSEM v.1.3.1 software [33]. Mapping of RNA-seq reads to genes (excluding introns) was performed using program Bowtie2 [30], with parameters provided by RSEM script. Data normalization and differential expression analysis was performed using functions of the DESeq2 v.1.38.3 [34] package in R with default parameters. Genes were considered differentially expressed if changes in the expression levels were not less than twofold [|log2(FC)| ≥ 1] and the adjusted *p*-value was ≤0.05.

Functional annotation of the assembled transcripts, including KEGG terms, were transferred from model organism *S. cerevisiae* S288c; new genes were annotated manually using NCBI NR database and KAAS annotation server [35].

KEGG terms enrichment was carried out with the clusterProfiler v.3.12.0 package in R programming language [36]; terms with adjusted *p*-value ≤ 0.05 were considered significantly enriched.

### 2.6. Nucleotide Sequence Accession Numbers

This BioProject has been deposited in GenBank under accession number PRJNA1187884. The sequences obtained in this project have been deposited in the NCBI Sequence Read Archive under the accession numbers SRR31406242-SRR31406247.

### 2.7. Characteristics of Morphological, Cultural, Physiological, and Biochemical Properties of Yeast

The strains were cultured in tubes in must at a temperature of (26 ± 0.5) °C. The shape and size of the cells were assessed by microscopic examination of the culture in the exponential phase of growth. The cell sizes were determined and analyzed using digital images using the Image Scope M computer program. The presence of surface growth and the structure of the sediment were determined visually in a 30-day culture. The nature of the sediments formed; the presence of a ring and a velum on the surface of the culture medium were visually assessed.

The ability of yeast to form spores was assessed based on the results of growth on Gorodkova’s solid medium (1.1% peptone, 0.5% NaCl, 0.25% glucose, 2% agar–agar, pH 7.3). An active culture of the studied yeast samples was seeded on the surface of the medium in Petri plates. The cultures were incubated at a temperature of (26 ± 0.5) °C and examined microscopically every three days for 5–6 weeks to detect the appearance of spores.

The ability of the strains to produce hydrogen sulfide was studied using a method using a dense nutrient medium BIGGY Agar [37].

The ability of the strains to produce acetic acid was assessed by the formation of a transparent halo surrounding the inoculation zone. The strains were cultured for 72 h at a temperature of (30 ± 0.5) °C on Chalk’s a dense medium with (0.3% yeast extract, 1% glucose, 0.3% calcium carbamate, 1.5% agar) [38]. The activity of the strain to produce acetic acid was assessed using the following scale: 0—halo 0–1 mm (very weak ability); 1—halo from 1 to 3 mm (low ability); 2—halo between 3 and 5 mm (medium ability); 3—halo 4–5 mm (high ability).

The acid and alcohol tolerance, cold and heat resistance, and sulfite resistance were assessed based on the growth reaction of yeast cells to the cultivation conditions on a synthetic YPD medium (1% yeast extract, 2% peptone, 2% glucose, pH 3.4). When assessing cold resistance, the cultures were incubated at a temperature of (10 ± 1) °C; for heat resistance at (37 ± 1) °C; and when assessing acid tolerance, at a temperature of (26 ± 1) °C and pH 2.6. When assessing sulfite resistance, the cultures were incubated at a temperature of (26 ± 1) °C and a mass concentration of total sulfur dioxide in the medium of 200 mg/L. When assessing alcohol tolerance, the cultures were incubated at a temperature of (26 ± 0.5) °C and an ethanol content of 10% *v*/*v* and 12% *v*/*v*. For a clearer identification of the yeast reaction to new conditions, micro-seeding was used to the initial number of cells in the medium of 8–30 × 10^3^ per mL. The tubes were inspected daily for 5 days. The growth reaction of the strains to the specified cultivation conditions (presence of growth, absence of growth) was visually noted.

### 2.8. The Ability of Yeast Strains to Ferment Malic Acid

The strains were cultured in a model medium (0.4% tartaric acid, 10% glucose, 10% fructose, 2% yeast peptone, 1% yeast extract, pH 3.8). Three versions of the media were prepared by adding L-malic acid to the model medium in concentrations of 2 g/L, 3 g/L, and 4 g/L; 100 mL of the medium was poured into sterile 200 mL flasks, sealed with cotton-gauze stoppers and the culture was added to a cell concentration of 2 × 10^6^ per mL. Cultivation was carried out at a temperature of (20 ± 1) °C until the end of fermentation. The clarified fermented must was decanted from the sediment and analyzed using high-performance liquid chromatography.

### 2.9. Evaluation of Fermentation Activity

Fermentation activity in laboratory conditions was assessed by the amount of carbon dioxide released during fermentation of pasteurized must from the Aligoté grape variety (mass concentration of sugars—235 g/L; titratable acids—4.3 g/L; total sulfur dioxide—87 mg/L; pH—3.4) in special flasks with fermentation locks. The seeding was carried out with a two-day culture in an active state to the initial concentration of cells of 2 × 10^6^ per mL. The flasks were kept in at a temperature of (17 ± 1) °C. The flasks were weighed daily for 30 days, determining the amount of carbon dioxide released during fermentation of must. Based on the values of three repetitions, the average value of the indicator was found and recalculated for a must volume of 100 mL.

### 2.10. Large-Scale Fermentation Procedure

The wines were prepared from one batch of grapes (50 kg) of the Rkatsiteli grape variety (mass concentration of sugars—195 g/L, titratable acids—5.7 g/L, pH—3.3) according to the following scheme: destemming; crushing; pressing; separation of must in an amount of no more than 600 mL/kg of grapes; sulfitation of must (70–75 mg/L of total sulfur dioxide); clarification of must for 24 h at a temperature of (10 ± 2) °C; addition of 3 g/L of L-malic acid to pH 3.0; inoculation with a dilution (3% *v*/*v*) of pure culture of the studied strains; fermentation at a temperature of (22 ± 2) °C; removal of wine material from yeast sediment; further fermentation until the density became less than 1.0 g/mL and self-clarification of wine; settling and removal from sediment; sulfitation (addition of 150 mg/L total sulfur dioxide). For inoculation, physiologically active yeast cultures were used (cell count 1.2 × 10^8^ per mL, budding cell count 42%, dead cells—0.5%).

### 2.11. Oenological Parameters Analysis

The mass concentration of sugars was determined using a DIN 12791/L50 hydrometer (Schneider, Germany), the pH and the mass concentration of titratable acids were determined using a universal ion meter I-160 (TD Avtomatika, Belarus) according to standard methods [39]. All measurements were performed in triplicate.

After the end of fermentation (day 30), the following parameters were assessed in wines: volume fraction of ethanol, mass concentration of organic acids, residual sugars, and pH. The quantitative measurement of organic acids was performed by high-performance liquid chromatography (HPLC). The samples were separated into individual substances using a Supelcodel C610H column (Sigma-Aldrich, Burlington, MA, USA) filled with a sorbent based on sulfited divinyl polystyrene (column size 300 × 7.8, sorbent grain size no more than 10.0 μm) on an LC20AD Shimadzu chromatograph (Kyoto, Japan) equipped with a spectrophotometric detector. An aqueous solution of orthophosphoric acid (1 g/L) was used as an eluent. The mass concentration of organic acids in the wine sample was determined according to preliminary calibration of the device using pure substance standards using the spectrometric detector of the system at 210 nm, taking into account the release time and spectral characteristics of each of the individual substances. In the case of the presence of suspended matter or insoluble particles during a visual assessment of a wine material sample, they were preliminarily separated using a centrifuge (rotor speed of at least 6–7 thousand rpm, duration—no more than 5–7 min). All measurements were performed in triplicate.

The concentration of ethyl carbamate and urea was determined using HPLC [40,41]. The sample derivatization reaction was conducted in chromatographic vials as follows: 0.4 mL of 9-xanthydrol solution (0.02 mol of 9-xanthydrol per liter in 1-propanol) was added to 0.6 mL of a sample or standard, and then 0.1 mL of hydrochloric acid (1.5 mol/L) was slowly added. To ensure the reaction reached completion, the solution was homogenized and held for 30 min in the dark before being injected into chromatographic system for analysis. Three repeated injections of standard solutions, at six concentrations, were used to generate calibration curves. The analysis was conducted on LC20AD Shimadzu chromatograph (Japan) with a quaternary solvent delivery system and a fluorescence detector. The flow rate was 1.0 mL/min, the oven temperature 30 °C, and the injection volume 20 μL. The excitation and emission wavelengths were 240 and 308 nm, respectively. Phase A was sodium acetate (pH 7.2, adjusted with acetic acid). Phase B was acetonitrile.

Organoleptic assessment of wines was carried out using a 100-point system [42].

### 2.12. Statistical Analysis

The experiments were repeated three times, and the results are presented as mean and standard deviation. Statistical analysis was performed using SPSS Statistics v. 17.0. Differences between the engineered strain and the parental strain were confirmed by Student’s t-test. Differences were considered statistically significant at *p*-value < 0.05.

## 3. Results and Discussion

### 3.1. Construction of a Yeast Strain with CAR1 Gene Deletion and Insertion of the MLF Cassette Using the CRISPR/Cas9 System

To create a strain with reduced ethyl carbamate production, we deleted the *CAR1* gene, which is responsible for the breakdown of arginine to urea. To achieve this gene deletion, we used the CRISPR/Cas9 system and designed a donor DNA fragment containing a cassette for the expression of malolactic enzyme and malate permease, flanked by the sequences located upstream and downstream of the *CAR1* gene. This DNA fragment allows for the replacement of the *CAR1* gene with the MLF cassette through homologous recombination.

**Figure 1:**
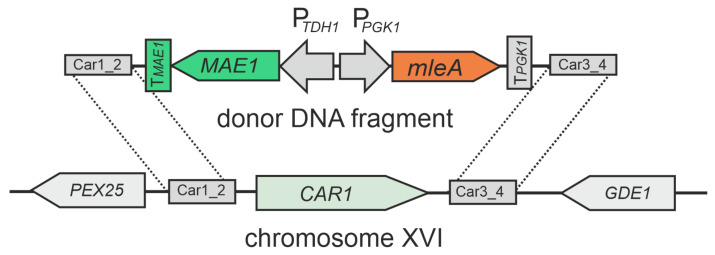
Scheme of replacement of *CAR1* gene with the MLF cassette. *mleA*—gene encoding malolactic enzyme from *O. oeni* K19-3; *MAE1*—gene of malate permease from *S. pombe* I-583 with its own terminator (T*_MAE1_*). T*_PGK1_*—terminator of the *PGK1* gene from *S. cerevisiae* I-329; P*_TDH1_*—promoter of the *TDH1* gene from *S. cerevisiae* I-329.

To express Cas9, which introduces a double-stranded DNA break, we used the plasmid pWS-amdS. The second plasmid, pWS82-CAR1-3, produced sgRNA directing Cas9 to the *CAR1* gene. Yeast strain I-328 was transformed with the appropriate fragments of these plasmids together with a linear donor DNA fragment containing the MLF gene expression cassette (see Materials and Methods for details). The replacement scheme is shown in Figure 1.

Yeast transformants were selected on a synthetic minimal SC medium containing acetamide (0.3%) as a nitrogen source instead of ammonium sulfate. Based on the frequency of occurrence of single-nucleotide polymorphisms, it was found that strain I-328 is a triploid (Figure S1). This means that upon integration, the cassette could replace one, two, or all three genomic copies of the *CAR1* locus. In the latter case, the transformant should lose Car1 arginase activity and exhibit a phenotype characterized by significant growth inhibition on the synthetic minimal medium SC + arg, where ammonium sulfate has been replaced by arginine as the sole nitrogen source [43]. Therefore, the selected transformants were tested for their ability to grow on solid SC + arg medium. As a result, several transformants with strong growth inhibition on this medium were identified, and one of these clones was selected for further analysis.

The replacement of the *CAR1* gene with the MLF cassette in the transformants was confirmed by PCR. Consequently, we selected the clone I-328 ∆CAR1_MLF, which was then cultured for several days in non-selective medium to eliminate the derivative of the pWS-amdS plasmid.

Finally, whole-genome sequencing of the *S. cerevisiae* I-328 ∆CAR1_MLF strain confirmed the deletion of all three genomic copies the *CAR1* gene and the insertion of the MLF expression cassette. No additional genomic rearrangements or point mutations were detected.

### 3.2. The Effect of Integration of the MLF Cassette in Place of the CAR1 Gene on the Transcriptome

We analyzed how the gene transcription profile changed in the modified strain I-328 ∆CAR1_MLF relative to the parental strain I-328. For transcriptomic analysis, must from the Aligoté variety was inoculated with the parental I-328 and I-328 ∆CAR1_MLF strain, with three replicates for each. RNA samples were isolated on the 7th day, after the completion of the active fermentation stage.

Transcriptome analysis based on RNA-Seq showed that a total of 5314 genes were expressed in at least one of the samples. Among them, there were 220 differently expressed genes (DEG) (log2FoldChange > 1, padj < 0.05), of which only 49 genes showed increased expression in the modified strain, while the remaining 171 showed decreased expression (Table S1).

Consistent with the absence of the *CAR1* gene, it was not detected in the transcriptome of the modified strain. On the other hand, the engineered strain I-328 ∆CAR1_MLF demonstrated the expression of both the malolactic enzyme and the malate permease genes, absent in the parental strain. The obtained results confirm the active expression of the MLF cassette in the engineered strain.

Among genes involved in the arginine metabolism, a 3.4-fold increase in transcription was detected for the *CAR2* gene encoding ornithine aminotransferase that converts ornithine produced from arginine into glutamate-5-semialdehyde. Elevated transcription of this gene in I-328 ∆CAR1_MLF strain probably reflects an increase in the intracellular arginine content caused by the *CAR1* deletion, which induced *CAR2* transcription via the regulators ArgR and Mcm1 [44]. Transcription of the arginine permease gene *CAN1* was decreased 1.4-fold, suggesting that arginine import into the cell was decreased. Transcription of the *DUR1,2* gene encoding urea amidolyase and *DUR3* gene encoding urea transporter decreased 1,4 and 2 times, respectively, which corresponds to the expected lower level of urea production.

Analysis of DEGs using the KEGG database (Table S1) indicated that the pathways with increased expression were thiamine metabolism (10 genes), vitamin B6 metabolism (5 genes), and transport (2 genes). The most significant increase in transcription was observed for genes encoding aquaporin-2 (15-fold), 4-amino-5-hydroxymethyl -2-methylpyrimidine phosphate synthase THI5 (12-fold), and mating factor alpha-1 (7-fold).

Genes with reduced expression were categorized into the following pathways: meiosis (10 genes), purine metabolism (6 genes), galactose metabolism (6 genes), fructose and mannose metabolism (4 genes), and structural proteins (4 genes). Notably, a decrease in expression levels was observed for genes encoding hexose transporters Hxt8, Hxt10, Hxt15, Hxt17, sugar transporter Stl1, general alpha-glucoside permease Mal11, oligo-1,6-glucosidase Ima1, alpha-glucosidase Mal32, and maltose fermentation regulatory protein Mal63. It is possible that imported malate may not only be decarboxylated but also partially directed into central metabolic pathways, for example, through the action of malate dehydrogenase, thereby reducing the use of other sugars.

Overall, the transcription profiling of strains I-328 ∆CAR1_MLF and I-328 did not reveal any significant changes in the transcription of the genes of the central metabolism.

### 3.3. Morphological, Cultural, Physiological and Biochemical Properties of Strains

A comparative analysis of the morphological and cultural properties of I-328 ∆CAR1_MLF and I-328 strains showed that the strains do not differ from each other in their main features (Table 2). No significant differences were found between the strains in the shape and size of the cells, as well as in the structure of the sediment formed. The cells of the strains at the stage of must fermentation had a round-ovoid and oval shape, as stated for the collection strain I-328. The yeast sediment at the end of fermentation was dusty and light; the wine clarified well, and surface growth was absent in both strains.

When studying the ability of yeast to sporulate, it was noted that the parental strain formed smooth round spores of 1–4 in an ascus. For the modified strain I-328 ∆CAR1_MLF, sporulation was not observed.

The inclusion of MLF genes did not affect the phenotype of the engineered strain, which, according to the obtained results, belongs to the sensitive (S) phenotype, like the parental strain. Also, no effect of genome editing on the physiological and biochemical properties of the strain was noted: both strains were characterized by an average ability to synthesize hydrogen sulfide and a weak ability to form acetic acid.

### 3.4. Functional Characteristics of the Modified and Parental Strains

**Figure 2:**
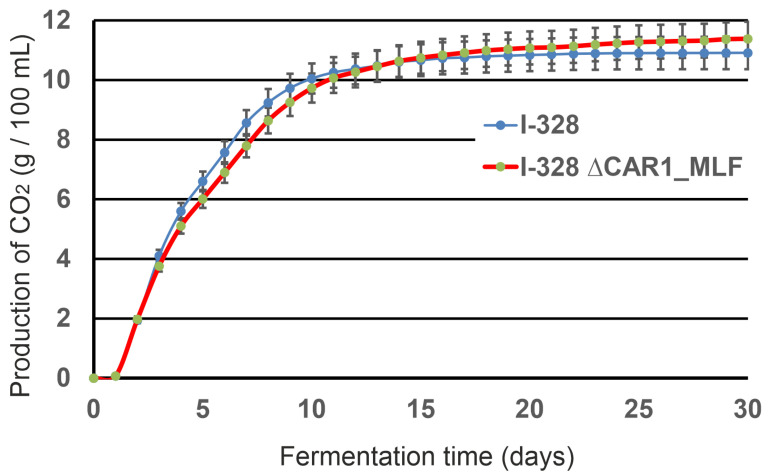
Fermentation activity of strains I-328 and I-328 ∆CAR1_MLF.

The fermentation activity of the I-328 and I-328 ∆CAR1_MLF strains was determined in an experiment on fermentation of pasteurized must. Comparison of the growth dynamics showed that the intensity of CO_2_ formation in the I-328 ∆CAR1_MLF strain was at the level of the parental I-328 strain (Figure 2). Thus, for the parental and modified strain, the amount of CO_2_ released in the active phase of fermentation for 15 days was 10.2 and 10.3 g/100 mL, respectively. This result shows that the replacement of the *CAR1* gene with the MLF cassette did not affect the fermentation activity.

Testing the resistance of the modified and original strains to stressful conditions of winemaking showed that both strains retained the ability to actively reproduce under different cultivation conditions for the same time periods as the original strain (Table S2). No significant differences were found between the studied strains. Analysis of the ability of yeast to adapt to individual abiotic factors showed that both strains demonstrated the highest sensitivity to ethanol at a concentration of 12% *v*/*v* and the least sensitivity to acidity of the environment, high temperatures, and sulfur dioxide.

The fermentation of L-malic acid by yeast was assessed on model media containing different amounts of malate (Table 3). It was found that when using the control strain, the malic acid content decreased by 21–24%. At the same time, the use of the modified strain I-328 ∆CAR1_MLF ensured a decrease in the malate content by 84–90%, which led to an increase in pH values. Although the residual glucose and fructose content in wine was higher in case of the I-328 ∆CAR1_MLF strain, both strains ensured the fermentation of sugars to the standard values for dry wines (less than 4.0 g/L). The higher level of residual sugars is consistent with the reduced transcription level of hexose transporters genes in the I-328 ∆CAR1_MLF strain. Genome editing did not affect the fermentation activity of yeast and the accumulation of ethanol. The mass concentration of volatile acids in the obtained wine samples was in trace amounts and did not exceed 0.1 g/L.

Similar functional characteristics were reported for the strain ML01, which was able to decarboxylate 5.5 g/L of malate and produce equimolar amounts of lactate without affecting the fermentation kinetics and production of ethanol [8]. As in the case of strain I-328 ∆CAR1_MLF, the residual sugar content in wine fermented with strain ML01 was higher than in the case of the corresponding parental strain [8].

To assess the impact of the studied yeast strains on the quality characteristics of the products, wines from Rkatsiteli grapes were prepared. Analysis of the obtained data confirmed that the introduction of the MLF genes into the genome of the I-328 strain does not affect the fermentation activity. Active fermentation of the must for both the control and modified strains was noted on the second day; the studied strains did not differ significantly in fermentation rate and ethanol production; the volume fraction of ethanol in the wines was 11.5 and 11.6% *v*/*v*/, respectively (Table 4).

Analysis of the acid profile showed that the wines did not differ in the content of citric, succinic, and acetic acids (Table 4). The use of the I-328 ∆CAR1_MLF strain enabled the MLF process at the fermentation stage, which resulted in a decrease in the content of malic acid by 90% and the production of an equimolar amount of lactic acid.

A tasting evaluation of the resulting wines showed that the sample prepared using the modified strain was characterized by a softer and more harmonious taste compared to the control, which was expressed in a higher tasting score—83 and 80 points, respectively.

A distinctive feature of the modified strain I-328 ∆CAR1_MLF was low urea production (1.8 times relative to the control), which resulted in a lower content of EC (Table 4). Consistent with other studies on deletion of the CAR1 gene [12,13,14,20], the results obtained confirm the effectiveness of arginase inactivation in reducing urea synthesis and EC content in wine.

## 4. Conclusions

Thus, for the first time, a triploid wine yeast strain was constructed, which simultaneously reduces the formation of ethyl carbamate and the level of malic acid in wine. This result was achieved by replacing the arginase gene *CAR1* with a cassette of genes that ensure the import of malate and its decarboxylation to lactate. Characterization of the obtained engineered strain using genomic, transcriptomic, and microbiological methods showed that genetic modification carried out did not lead to significant changes in its winemaking-relevant properties compared to the parental strain. The use of the modified strain I-328 ∆CAR1_MLF made it possible to reduce the level of ethyl carbamate in wine by half and reduce the content of malic acid by ten times. Tasting evaluation showed that the resulting wines had a softer and more harmonious taste compared to the wine obtained using the original strain. Thus, the new strain I-328 ∆CAR1_MLF is promising for further use in winemaking.

## Figures and Tables

**Table 1:** Oligonucleotides used in this work.

Primer	Sequence (5′-3′)
Car_1F	AACCGTGTAGGCAAAACTGGAC
Car_2R	CTCGAGTCGCGACCCGGGAGACAGTATGCGTAGCTTTACCA
Car_3R	CCCGGGTCGCGACTCGAGATCATCATCCCTTTTATCAAAATAAGCA
Car_4R	AGCGCTAAGAAGTGGAATTTATT
maeF	AAAACAAAATGGGTGAACTCAAGGAAAT
maeR	ATCCCGGGAGTACAATAAAGATACAGAA
PtdhF	ATTCGCGATTAGTAAAAATGTGCGCACCA
PtdhR	TCACCCATTTTGTTTTGTGTGTAAATTT
PpgkF	TATCGCGAACGCACAGATATTATAACAT
PpgkR	TCTGTCATTGTTTTATATTTGTTGTAAA
mleF	ATAAAACAATGACAGATCCAGTAAGTAT
mleR	AATTCAATTTAGTATTTCGGCTCCCACT
TpgkF	AATACTAAATTGAATTGAATTGAAATCG
TpgkR	ATCTCGAGAGGCATTAAAAGAGGAGCGA

**Table 2:** Morphological and cultural properties of strains.

Strain	Cell Morphology	Average Cell Size, µm	Sediment	Ring *	Velum *
I-328	Round-ovoid; elongated and oval ones are also found	8.43 × 5.84	Dusty	No	No
I-328 ∆CAR1_MLF	Round-ovoid; elongated and oval ones are also found	8.55 × 6.01	Dusty	No	No

* on the surface of the wine.

**Table 3:** Malate consumption in a model experiment.

Strain	Initial Content of L-Malic Acid (g/L)	∆ рН	Final Content					
			Malic Acid (g/L)	Lactic Acid (g/L)	Acetic Acid (g/L)	Glucose (g/L)	Fructose (g/L)	Ethanol (%, *v*/*v*)
I-328	2	+0.06	1.56 ± 0.02	0.31 ± 0.03	0.03 ± 0.01	0.74 ± 0.1	0.18 ± 0.1	9.70 ± 0.05
	3	+0.11	2.39 ± 0.01	0.42 ± 0.02	0.04 ± 0.01	0.64 ± 0.1	0.21 ± 0.1	9.72 ± 0.05
	4	+0.11	3.07 ± 0.03	0.65 ± 0.02	0.05 ± 0.01	0.68 ± 0.1	0.20 ± 0.1	9.76 ± 0.05
I-328 ∆CAR1_MLF	2	+0.15	0.24 ± 0.02	1.20 ± 0.01	0.03 ± 0.01	2.18 ± 0.1	0.70 ± 0.1	9.74 ± 0.05
	3	+0.19	0.48 ± 0.01	1.70 ± 0.02	0.03 ± 0.01	2.06 ± 0.1	0.67 ± 0.1	9.87 ± 0.05
	4	+0.21	0.40 ± 0.01	2.44 ± 0.02	0.03 ± 0.01	2.10 ± 0.1	0.66 ± 0.1	9.50 ± 0.05

**Table 4:** Chemical composition of wines prepared using I-328 and I-328 ∆CAR1_MLF strains.

Characteristic	I-328	I-328 ∆CAR1_MLF
Glucose, g/L	1.1 ± 0.1	1.3 ± 0.1
Fructose, g/L	1.0 ± 0.1	1.2 ± 0.1
Ethanol, % (*v*/*v*)	11.5 ± 0.05	11.6 ± 0.05
Citric acid, g/L	0.76 ± 0.05	0.74 ± 0.05
Succinic acid, g/L	0.28 ± 0.05	0.32 ± 0.05
Acetic acid, g/L	0.26 ± 0.05	0.25 ± 0.05
Glycerol, g/L	7.0 ± 0.05	7.1 ± 0.05
Titratable acidity, g/L	9.45 ± 0.05	8.66 ± 0.05 *
Malic acid, g/L	3.98 ± 0.05	0.31 ± 0.05 *
Lactic acid, g/L	0.66 ± 0.05	3.02 ± 0.05 *
Urea, mg/L	6.12 ± 0.05	3.45 ± 0.05 *
Ethyl carbamate, µg/L	15.4 ± 0.05	7.2 ± 0.05 *
рН	3.03 ± 0.05	3.1 ± 0.05

*—statistically significant differences (*p* < 0.05).

## Data Availability

The data presented in this study are contained within the article. The sequences obtained in this work have been deposited in the NCBI Sequence Read Archive under the accession numbers SRR31406242-SRR31406247. [NCBI Sequence Read Archive] [https://www.ncbi.nlm.nih.gov/sra/SRR31406242] (accessed on 25 November 2024) [SRR31406242-SRR31406247].

## Supplementary Materials

The following supporting information can be downloaded at: https://www.mdpi.com/article/10.3390/foods14010054/s1, Figure S1: Allele frequencies identified at SNP points in the genome according to sequencing reads; Table S1: The results of the differential gene expression analysis; Table S2: Resistance of strains to stressful cultivation conditions.
