# Supplementary material for: Development of a Wine Yeast Strain Capable of Malolactic Fermentation and Reducing the Ethyl Carbamate Content in Wine
Source: Foods. 2024 Dec 27;14(1):54. doi: 10.3390/foods14010054 (PMC11719754; doi:10.3390/foods14010054)
Supplement: Supplementary file 1 [file foods-14-00054-s001.zip › Figure_S1.pdf]

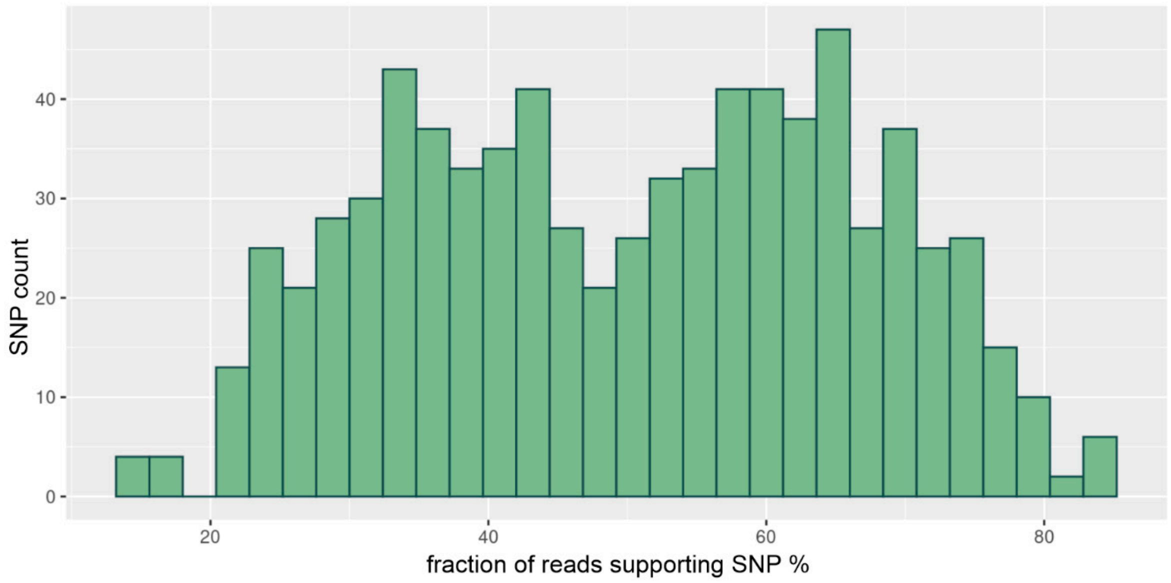

Figure S1. Allele frequencies identified at SNP points in the genome according to sequencing reads.

The distribution shows that the genome is triploid, with SNP frequency peaks around 33% and 67% of particular allele frequency.
