# Supplementary material for: Development of a Wine Yeast Strain Capable of Malolactic Fermentation and Reducing the Ethyl Carbamate Content in Wine
Source: Foods. 2024 Dec 27;14(1):54. doi: 10.3390/foods14010054 (PMC11719754; doi:10.3390/foods14010054)
Supplement: Supplementary file 1 [file foods-14-00054-s001.zip › Table S2.pdf]

Table S2. Resistance of strains to stressful cultivation conditions.

Strain	Start of growth, days after inoculation					
	Temperature 10 °C	Temperature 37 °C	pH 2.6	Concentration of sulfurous acid 200 mg/L	Ethanol 10 % (v/v)	Ethanol 12 % (v/v)
I-328	5	1	1	1	4	No growth
I-328 ΔCAR1_MLF	5	1	1	2	4	No growth
